# Play together with grandchildren: a potential useful strategy for promoting healthy aging suggested by the evidence of 1,293 Chinese older adults

**DOI:** 10.1186/s12877-026-07465-z

**Published:** 2026-04-14

**Authors:** Hongbiao Chen, Yuan Fang, Fengjuan Chen, Qinyi Wu, Siyu Chen, Shaojian Xu, Lixian Su, He Cao, Zixin Wang

**Affiliations:** 1 Longhua District Centre for Disease Control and Prevention, Shenzhen, Guangdong China; 2 Department of Health and Physical Education, The Education University of Hong Kong, Hong Kong, China; 3 Dapeng District Centre for Disease Control and Prevention, Shenzhen, Guangdong China; 4 Centre for Health Behaviours Research, JC School of Public Health and Primary Care, The Chinese University of Hong Kong, Hong Kong, China; 5 Futian District Maternal and Child Health Hospital, Shenzhen, Guangdong China

**Keywords:** Attitude towards aging, Older people, Family harmony, Intergeneration, Loneliness, Physical activity

## Abstract

**Background:**

China is one of the countries with the most serious aging problem. Although intergenerational activities are evident to benefit older adults’ wellbeing, the co-participation in physical activity (Co-PA) with grandchildren and its relationships with psychosocial outcomes in older people has been rarely reported under the Chinese context. This study investigated the level of different types of Co-PA with grandchildren among older adults aged 60 years or above and the associations between Co-PA levels and loneliness, attitudes toward aging, and family harmony.

**Methods:**

This is a secondary analysis of a cross-sectional survey conducted between August and October 2024 in Shenzhen, China. Participants of the original survey were residents aged ≥ 60 years with administrative health records in community health centers. Multistage random sampling was used. Ten community health centers were first randomly selected. Each selected community center then randomly selected 200 administrative health records of residents aged ≥ 60 years. A sub-sample of 1,293 participants with at least one grandchild aged < 18 years were included in the analysis. Multilevel (level 1: individual participants; level 2: community health centers) multivariable linear regression models were fitted.

**Results:**

The proportion of participants who performed the following Co-PA with grandchildren at least once per week was 69.7% for walking and cycling in the free time, 48.6% for playing sports, 46.3% for going to park, playground or beach, 41.8% for going to indoor recreational centre, and 35.4% for participating in sports competitions. Higher frequency of performing the aforementioned types of Co-PA and any type of Co-PA were associated with more positive attitudes toward aging (adjusted β: 0.98 to 1.47, all *p* < 0.001) and better family harmony (adjusted β: 0.27 to 0.56, all *p* < 0.001). However, the association between Co-PA and loneliness was non-significant.

**Conclusions:**

This study supports that intergenerational Co-PA may serve as a new strategy for promoting healthy aging in Chinese older people. Future efforts may be put on development of proper Co-PA programs, which may benefit both elders and children.

**Clinical trial number:**

Not applicable.

**Supplementary Information:**

The online version contains supplementary material available at 10.1186/s12877-026-07465-z.

## Background

Globally, the number of people who reached 60 years will increase from 1 billion in 2025 to 2.1 billion by 2050 [1]. In China, 21.1% of the population were aged 60 years old or above in 2023 [2]. Facilitating healthy aging becomes increasingly important. Previous studies suggested an urgent need to improve mental health among older adults. Across countries, 27.2% of suicide deaths occurred in people aged 60 years or above [3], while one quarter of older adults are affected by loneliness and social isolation [4]. Similar findings were observed in China, as 23.8% of older adults experienced loneliness in the country [5]. Loneliness may negatively affect older adults’ well-being, which could lead to a decrease in cognitive function or mobility, and an increase in mortality [6]. Furthermore, the attitudes towards aging, which were conceptualized as “being different by virtue of being older” and “judgments based on an individual’s age” [7], were more negative among older adults in China than their counterparts in western countries. This may be due to the Chinese traditional belief that the older adults are expected not to make efforts to do anything. Previous studies suggested that older adults with more negative attitudes towards aging were more likely to have worse health outcomes [8, 9]. The physical and social home environment plays an important role for among older people [10]. They are deemed as health markers to assess psychosocial health in this population [11, 12].

Participation in physical activity (PA) across the lifespan is essential for maintaining functional independence and preventing chronic disease later in life [13, 14]. The World Health Organization (WHO) recommends that adults aged 65 years or above to perform 150–300 min of moderate-intensity aerobic activity or 75–150 min of vigorous-intensity aerobic activity per week [15]. Regular PA could reduce the risk of cardiovascular disease, diabetes, and stroke, and improve mental health and quality of life among older adults [16]. However, at a global scale, 31% of older adults fail to meet the WHO recommendations; in contrary, the prevalence of physical inactivity has increased significantly after the age of 60 years [17].

Co-participation in PA (Co-PA) refers to two or more family members participating in PA together, such as grandparents and grandchildren [18]. According to a recently published scoping review on Co-PA [19], Co-PA is primarily understood as a one-dimensional variable linked to children’s PA levels or sport participation rates; and additionally, is now deemed as a dynamic process influencing the developmental outcomes of both children and the adults engaging in the activity with them. The second perspective emphasizes the potential benefits of Co-PA in family well-being beyond simply measuring PA levels or sports participation as isolated variables. Preliminary evidence suggested that Co-PA had positive effects on parent-child relationship. It was reported that experience of high-quality time with children during Co-PA programs significantly enhanced parent-child bonding and the development of shared interests [20–22]. In accordance, positive affect such as praise, meaningful conversations, family warmth, were also notably enhanced [23, 24].

In the Chinese context, family harmony which stands for the positivity of family relationships in the Chinese cultures [25], is considered as one of three core values in the Chinese families (i.e., harmony, happiness, and health, 3 H) [26]. In China, intergeneration mutual care is common. Previous studies reported that more than 80% of Chinese older adults were living with other family members [27–29]. In a recent investigation, nearly half of surveyed older people spent 40 h per week to take care of their grandchildren [30]. Furthermore, evidence demonstrated that Chinese grandparents actively participated in daily care of their grandchildren, providing many opportunities for intergenerational interaction. During the daily interaction, support and bonding with family members would cultivate positive attitude toward aging and reduce the loneliness among older adults [6]. Grandparent-grandchildren Co-PA is one of these interactions [30–32].

Previous studies mainly focused on parent-child Co-PA in the literature [33, 34], whereas very few studies reported the impacts of intergenerational Co-PA on physical, emotional, and social well-beings among older adults, especially in China. To address the knowledge gaps, this study investigated the level of any types and specific type of Co-PA with grandchildren among older adults aged 60 years or above. In addition, association between Co-PA with grandchildren and perceived loneliness, attitudes toward aging, and family harmony were also investigated. We hypothesized that higher levels of Co-PA with grandchildren would be associated with lower loneliness, but more positive attitudes to aging and better family harmony. 

## Methods

### Study design and study population

This is a secondary analysis of a cross-sectional survey conducted among people aged 60 years or above in Shenzhen between August and October 2024. Shenzhen is a city and special economic zone in the southern Chinese province of Guangdong, bordering Hong Kong to the south. Shenzhen is one of the four most developed cities (Beijing, Shanghai, Guangzhou, and Shenzhen) in mainland China, with a population of 17 million in 2021. This study was based on a sub-sample of older adults who had at least one grandchild under the age of 18 years at the time of the survey. In 2024, 60.8% of Chinese residents entered college or university at age 18 [35]. Most of the university or college students were living in the dormitories provided by the schools. It is less likely that they will have Co-PA with their grandparents. Therefore, we excluded subjects with all grandchildren aged 18 years or above in this study. 

### Participants and data collection

The inclusion criteria of participants in the original survey were: (1) people aged 60 years or above, (2) living in Shenzhen at the time of the survey, and (3) had established administrative health records in community health centers. Since 2014, all Chinese residents who have lived in the same region for more than six months, regardless of age or permanent residency should establish administrative health records in local community health centers. The health records contain basic sociodemographic information, children development indicators, information related to pregnancy and childbirth (for women), chronic disease diagnosis and management, and vaccination history.

The multistage random sampling approach was used. In the first stage, names of all community health centers in Shenzhen (926 in total) were entered in an Excel spreadsheet. Using the function of “selecting random cells”, ten community health centers were randomly selected. Within each selected community health center, 200 residents were randomly selected from administrative health records of people aged 60 years or above. Staff in the selected health centers approached prospective participants through telephone at different timeslots during weekdays and weekends, briefed them about the study, and invited them to visit the community health center to complete a face-to-face interview. On site, staff of the community center obtained their written informed consent, and performed the interview in Mandarin or Cantonese which took about 30 min to complete. A cash coupon of CNY25 (US$3.5) was given to each participant after completion of the interview. Ethical approval was obtained from the Longhua District Center for Disease Control and Prevention (CDC) (reference: 2024008). 

### Measurements

#### Development of the questionnaire

The study questionnaire was designed by a panel consisting of epidemiologists, behavioral health experts, sports scientists and CDC employees (Supplementary Material 1). To ensure clarity and readability, the questionnaire was pilot tested among ten older adults. All participants in the pilot testing found the questionnaire easy to understand. Based on their feedback, the panel made final adjustments to the questionnaire. Data of these ten older adults were not included in data analysis.

#### Background characteristics

Participants reported socio-demographic characteristics, including age, sex, education level, marital status, occupation, income, and residence registration. Information related to self-reported history of chronic conditions (e.g., hypertension, chronic lung disease, and diabetes) and severe acute respiratory syndrome coronavirus 2 (SARS-CoV-2) infection were also recorded.

#### Co-PA with grandchildren

Levels of Co-PA were measured by a validated tool developed by Hnatiuk et al. (2017) [36] with some modification. We replaced the word “children” in the original tool with “grandchildren”. The tool has 5 items measuring the frequency of Co-PA including: (1) Walking or cycling with their grandchildren in their free time, (2) Playing sport with their grandchildren, (3) Participating in sport competition with their grandchildren, (4) Going to the park, playground, beach or similar with their grandchildren, and (5) Going to an indoor recreation center with their grandchildren. The response categories were: 1 = never, 2 = no more than once per month, 3 = 2–3 days per month, 4 = one day per week, and 5 = at least two days per week. A higher score of the item or the scale indicated a higher level of Co-PA with grandchildren.

#### Loneliness, family harmony and attitudes toward aging

Loneliness was measured using the validated Chinese version of the 3-item UCLA Loneliness Scale [37]. The tool consists of three items assessing different aspects of loneliness in the past 2 weeks, including “How often do you feel that you lack companionship?”, “How often do you feel left out?”, and “How often do you feel isolated from others?”. Response categories range from 1 (“hardly ever or never”) to 3 (“often”), with a higher score indicating a higher level of loneliness. This concise tool has demonstrated good structural validity (CFI = 1.0, RMSEA = 0.00) and internal consistency (Cronbach’s α = 0.87).

Family harmony was measured using the validated Chinese version of the 5-item Family Harmony Scale (FHS) [38]. The FHS measured five key aspects of family harmony, including effective communication, conflict resolution, mutual tolerance, family identity, and quality time spent with family. Each item was rated on a 5-point Likert scale, ranging from 1 = strongly disagree to 5 = strongly agree. The FHS has shown good internal consistency (Cronbach’s α > 0.80) and robust psychometric properties, making it appropriate for use in large-scale epidemiological studies.

Attitudes toward aging were assessed using the Physical Change Subscale of the validated Chinese version of the 24-item Attitudes to Aging Questionnaire (AAQ) [39]. The Physical Change Subscale has 8 items (example items include “Growing old is easier than I thought.”, “It’s important to exercise at any age”). Each item is rated on a 5-point Likert scale (1 = “strongly disagree” to 5 = “strongly agree”). Higher scores in this subscale reflect more positive attitudes toward aging. The subscale demonstrated high internal consistency (Cronbach’s α = 0.79) and validity across diverse older populations.

### Statistical analysis

All statistical analyses were conducted using SPSS version 29.0.2.0 (IBM Corp, Armonk, NY, USA). Descriptive statistics of all studied variables were presented. Multilevel linear regression models (level 1: individual participants; level 2: community health centers) were used to analyze factors associated with the dependent variables. The individual participants (level 1) were nested within community health centers (level 2) in the analyses. Random intercept models were used to allow the intercept of the regression model to vary across community health centers, which could account for intra-cluster correlation within nested data. Multilevel regression models are commonly used in studies with similar sampling methods [40–42]. Using scores of the 3-item UCLA Loneliness Scale, the FHS, and the Physical Change Subscale as dependent variables, univariate multilevel linear regression models were used to assess the associations between background characteristics and the dependent variables. Multilevel multivariable linear regression models were then conducted to investigate the association between the independent variables of interest (overall Co-PA level and level of a specific type of Co-PA) and the dependent variables after adjusting for significant background characteristics. Unadjusted and adjusted regression coefficients (β) and their 95% confidence interval (CI) were obtained. Statistical significance was set at *p* < 0.05. 

## Results

### Background characteristics of the participants

Among 2,000 people aged 60 years or above approached by the research team, 74 had migrated to other cities at the time of this study, 370 refused to participate in the study due to lack of time and other tangible reasons, and 1,556 completed the face-to-face interviews. The data analysis was performed among 1,293 participants who had at least one grandchild under the age of 18 years. The mean age of the participants was 66.6 years (standard deviation [SD] = 5.2). Majority of the 1,293 participants were 60–70 years old (80.8%), female (60.9%), married (91.3%), living with the grandchildren (59.8%), main caregivers of the grandchildren (72.2%), without tertiary education (84.3%), and with a monthly income less than CNY5,000 (US$689). Among the participants, 9.9% and 9.3% reported smoking and binge drinking in the past year, 67.1% had any chronic conditions, and 94% reported a history of confirmed SARS-CoV-2 infection. (Table 1).

**Table 1:** Descriptive statistics of background characteristics, Co-PA and dependent variables (*n* = 1,293)

Descriptive results	*n*	%
Background characteristics		
Age group, years		
60–64	485	37.5
65–70	560	43.3
71–75	154	11.9
≥75	94	7.3
Sex assigned at birth		
Male	505	39.1
Female	788	60.9
Education level		
Primary or below	340	26.3
Secondary	750	58.0
Tertiary or above	203	15.7
Marital status		
Currently single	113	8.7
Married or cohabited with a partner	1180	91.3
Employment status		
Retired	1047	81.0
Full-time/part-time/freelance/other	236	19.0
Monthly income		
≤CNY¥4,999 (US$ 689)	713	55.1
CNY¥5,000–9,999 (US$ 690–1379)	188	14.6
≥CNY¥10,000 (US$ 1379)	392	30.3
Being main caregiver of the grandchild		
No	360	27.8
Yes	933	72.2
Living with other household members, Yes		
Spouse	775	60.0
Children	964	74.6
Grandchildren	773	59.8
Other relatives	22	1.7
Domestic helpers	13	1.0
Others	21	1.6
Smoking in the past year		
No	1165	90.1
Yes	128	9.9
Binge drinking in the past year		
No	1173	90.7
Yes	120	9.3
Presence of chronic conditions		
Hypertension	585	45.2
Other cardiovascular diseases	203	15.7
Chronic lung diseases	61	4.7
Chronic liver diseases	47	3.6
Chronic kidney diseases	62	4.8
Diabetes mellitus	283	21.9
Other diseases	92	7.1
Any of above	868	67.1
History of confirmed SARS-CoV-2 infection		
No	78	6.0
Yes	1215	94.0
Co-PA levels (Cronbach’s α: 0.91)		
Walking or cycling with their grandchildren in their free time		
Never	231	17.9
<1 day per month	133	10.3
2–3 days per month	157	12.1
1 day per week	321	24.8
>1 days per week	451	34.9
Item score, mean (SD)	3.5	1.5
Playing sport with their grandchildren		
Never	360	27.8
<1 day per month	148	11.4
2–3 days per month	157	12.1
1 day per week	304	23.5
>1 days per week	324	25.1
Item score, mean (SD)	3.0	1.6
Participating in sport competition with their grandchildren		
Never	581	44.9
<1 day per month	146	11.3
2–3 days per month	109	8.4
1 day per week	245	18.9
>1 days per week	212	16.4
Item score, mean (SD)	2.5	1.6
Going to the park, playground, beach or similar with their grandchildren		
Never	288	22.3
<1 day per month	206	15.9
2–3 days per month	201	15.5
1 day per week	287	22.2
>1 days per week	311	24.1
Item score, mean (SD)	3.1	1.5
Going to an indoor recreation centre with their grandchildren		
Never	384	29.7
<1 day per month	201	15.5
2–3 days per month	168	13.0
1 day per week	291	22.5
>1 days per week	249	19.3
Item score, mean (SD)	2.9	1.5
Co-PA Scale ^1^		
Mean (SD) ^2^	3.0	1.3
Dependent variables		
The Physical Change Subscale of the Attitudes toward Aging Questionnaire (AAQ) ^3^		
Scale score, mean (SD)	31.0	6.4
The 3-item UCLA Loneliness Scale ^4^		
Scale score, mean (SD)	4.3	1.6
The Family Harmony Scale ^5^		
Scale score, mean (SD)	20.9	4.1

^1^ Co-PA scale, 5 items, Cronbach’s α: 0.91

^2^ Mean score was calculated by summing up 5 individual item scores and divided by number of items

^3^ The Physical Change Subscale of AAQ, 8 items, range of score: 8–40, Cronbach’s α in this study sample: 0.92

^4^ The 3-item UCLA Loneliness Scale, 3 items, range of score: 3–9, Cronbach’s α in this study sample: 0.79

^5^ The Family Harmony Scale, 5 items, range of score: 5–25, Cronbach’s α in this study sample: 0.97

### Co-PA with grandchildren

Walking and cycling in the free time was the most common type of Co-PA with grandchildren, as 69.7% of participants performed it at least one day per week. The proportion of participants who performed the following type of Co-PA with grandchildren at least once per week was 48.6% for playing sports, 46.3% for going to park, playground or beach, 41.8% for going to indoor recreational centre, and 35.4% for participating in sports competitions. The mean (SD) for item and scale score related to Co-PA were presented in Table 1.

### Descriptive statistics of loneliness, family harmony, and attitudes toward aging

The mean scores (SD) were 31.0 (SD = 6.4) for the Physical Change Subscale of AAQ, 4.3 (SD = 1.6) for the 3-item UCLA Loneliness Scale, and 20.9 (SD = 4.1) for the Family Harmony Scale, respectively. The Cronbach’s alphas for these scales in this study sample ranged from 0.79 and 0.97 (Table 1).

### Associations between background characteristics and dependent variables

Being non-smokers in the past year was associated with more positive attitudes toward aging, better family harmony, and lower level of loneliness. Higher monthly income level was associated with more positive attitudes toward aging and better family harmony. Having higher education level was associated with more negative attitudes toward aging, but a lower level of loneliness. Living with grandchildren was associated with lower level of loneliness and better family harmony. In addition, living with spouse was associated with more positive attitudes toward aging and better family harmony (Table 2).

**Table 2:** Association between background information and psychosocial outcomes

	Attitudes to Aging		Loneliness		Family harmony	
	Unadjusted β (95% CI)	*P* value	Unadjusted β (95% CI)	*P* value	Unadjusted β (95% CI)	*P* value
Age group, years						
60–64	Reference		Reference		Reference	
65–70	0.17 (-0.63, 0.98)	0.67	-0.10 (-0.29, 0.10)	0.34	0.13 (-0.39, 0.65)	0.62
71–75	0.18, (-0.80, 1.16)	0.72	0.23 (-0.02, 0.47)	0.07	0.22 (-0.42, 0.86)	0.49
≥75	-0.39 (-1.82, 1.03)	0.59	-0.14 (-0.49, 0.21)	0.43	-0.08 (-0.99, 0.84)	0.87
Sex assigned at birth						
Male	Reference		Reference		Reference	
Female	0.30 (-0.41, 1.01)	0.41	-0.09 (-0.26, 0.08)	0.31	0.29 (-0.16, 0.75)	0.21
Education level						
Primary or below	Reference		Reference		Reference	
Secondary	-0.84 (-1.65, -0.03)	0.04	-0.18 (-0.38, 0.02)	0.08	-0.18 (-0.70, 0.35)	0.51
Tertiary or above	0.02 (-1.09, 1.13)	0.97	-0.52 (-0.79, -0.25)	< 0.001	-0.24 (-0.96, 0.47)	0.51
Marital status						
Currently single	Reference		Reference		Reference	
Married or cohabited	-0.50 (-1.72, 0.72)	0.43	0.12 (-0.18, 0.42)	0.42	-0.25 (-1.03, 0.54)	0.53
Employment status						
Retired	Reference		Reference		Reference	
Full-time/part-time/freelance/other	-0.01 (-0.90, 0.87)	0.98	0.10 (-0.12, 0.31)	0.38	0.28 (-0.28, 0.85)	0.33
Monthly income						
≤4,999 RMB (US$ 689)	Reference		Reference		Reference	
5,000–9,999 RMB (US$ 690–1379)	0.96 (-0.06, 1.98)	0.06	-0.24 (-0.49, 0.01)	0.06	0.01 (-0.65, 0.66)	0.98
≥10,000 RMB (US$ 1379)	0.99 (0.21, 1.78)	0.01	0.14 (-0.06, 0.33)	0.17	0.73 (0.22, 1.23)	0.01
Living with other household members, Yes						
Spouse	1.07 (0.37, 1.78)	0.003	-0.14 (-0.32, 0.03)	0.10	0.79 (0.34, 1.24)	< 0.001
Children	0.22 (-0.57, 1.02)	0.59	-0.14 (-0.34, 0.06)	0.16	0.41 (-0.11, 0.92)	0.12
Grandchildren	0.30 (-0.40, 1.01)	0.40	-0.24 (-0.42, -0.07)	0.01	0.79 (0.33, 1.24)	< 0.001
Other relatives	-2.54 (-5.20, 0.13)	0.06	-0.43 (-1.09, 0.22)	0.20	-2.28 (-4.00, -0.57)	0.01
Domestic helpers	0.75 (-2.70, 4.20)	0.67	-0.07 (-0.91, 0.78)	0.88	1.07 (-1.16, 3.29)	0.35
Others	0.90 (-1.84, 3.63)	0.52	-0.49 (-1.16, 0.19)	0.16	1.74 (-0.01, 3.50)	0.051
Being main caregiver of the grandchild						
No	Reference		Reference		Reference	
Yes	-0.88 (-1.65, -0.10)	0.03	-0.21 (-0.40, -0.02)	0.03	-0.14 (-0.64, 0.37)	0.60
Lifestyles and health conditions						
Smoking in the past year						
No	Reference		Reference		Reference	
Yes	-1.19 (-2.35, -0.04)	0.04	0.31 (0.03, 0.59)	0.03	-0.95 (-1.69, -0.21)	0.01
Binge drinking in the past year						
No	Reference		Reference		Reference	
Yes	-0.49 (-1.68, 0.71)	0.42	0.27 (-0.02, 0.57)	0.07	-0.76 (-1.52, 0.01)	0.053
Presence of any chronic conditions						
No	Reference		Reference		Reference	
Yes	0.04 (-0.69, 0.77)	0.92	-0.09 (-0.27, 0.08)	0.30	-0.07 (-0.54, 0.39)	0.76
History of confirmed SARS-CoV-2 infection						
No	Reference		Reference		Reference	
Yes	0.77 (0.00, 1.53)	0.05	0.15 (-0.04, 0.34)	0.11	0.21 (-0.29, 0.70)	0.41

*β* regression coefficient, *CI* confidence interval

### Association between five types of Co-PA levels and psychosocial outcomes

After adjustment of significant background characteristics, higher frequency of walking or cycling with grandchildren in the free time (adjusted β: 1.17 & 0.56, both *p* < 0.001), playing sports with grandchildren (adjusted β: 1.04 & 0.33, both *p* < 0.001), participating sport competition with grandchildren (adjusted β: 0.98 & 0.27, *p* < 0.001), and going to outdoor (adjusted β: 1.08 & 0.39, both *p* < 0.001) and indoor sports facilities (adjusted β: 1.00 & 0.31, both *p* < 0.001) were associated with more positive attitudes of aging and better family harmony. In addition, positive and significant associations between overall Co-PA level and the aforementioned outcomes were also observed (adjusted β: 1.47 & 0.50, both *p* < 0.001). However, the associations between frequency of a specific type of Co-PA or overall Co-PA level and loneliness were not statistically significant (Table 3).

**Table 3:** Association between participants’ levels of five Co-PA types with their grandchildren and psychosocial outcomes

Co-PA levels	Attitudes to Aging		Loneliness		Family harmony	
	Adjusted β (95%CI)	*p* values	Adjusted β (95%CI)	*p* values	Adjusted β (95%CI)	*p* values
Walking or cycling in the free time	1.17 (0.94, 1.43)	< 0.001	-0.04 (-0.10, 0.02)	0.22	0.56 (0.42, 0.71)	< 0.001
Playing sport	1.04 (0.82, 1.26)	< 0.001	-0.02 (-0.08, 0.04)	0.50	0.33 (0.18, 0.47)	< 0.001
Participating sport competition	0.98 (0.77, 1.20)	< 0.001	0.05 (-0.01, 0.11)	0.07	0.27 (0.13, 0.41)	< 0.001
Going to the park, playground, beach or similar	1.08 (0.85, 1.32)	< 0.001	0.001 (-0.06, 0.06)	0.98	0.39 (0.23, 0.52)	< 0.001
Going to an indoor recreation centre	1.00 (0.78, 1.22)	< 0.001	0.03 (-0.03, 0.08)	0.35	0.31 (0.16, 0.45)	< 0.001
Average score	1.47 (1.21, 1.73)	< 0.001	0.01 (-0.06, 0.07)	0.82	0.50 (0.33, 0.67)	< 0.001

Adjusted β: adjusted regression coefficient with adjustment of background covariates listed in Table 2

*CI* confidence interval

## Discussion

This is one of the first studies exploring the level of Co-PA with grandchildren among older adults in China. Positive and significant correlations between Co-PA and psychosocial outcomes (i.e., positive attitudes toward aging and family harmony) were also found by this study, which suggested that Co-PA might be a new strategy to improve intergenerational family relationship and aging-specific well-being in older adults. A relatively large study sample recruited through multi-stage random sampling is another strength of this study.

The role played by Chinese older people in the family system is different from their counterparts in western countries [43]. Majority of the Chinese older people provide care, guidance and economic contribution to their grandchildren, especially to those who are under 18 years old. Our findings supported this phenomenon in Chinese older people, as 74.6% of participants reported to live with their children and 72.2% of them were the main caregivers of their grandchildren. As the one of the three most important Chinese family values [26], family harmony which stands for the positivity of family relationships in the Chinese cultures [25]. Therefore, a good family atmosphere benefits this intergenerational relationship in a Chinese family system. In terms of this, a prevalent high level of family harmony was perceived in the older participants of this study.

Compared with the previous studies targeting older people in other countries [8, 44], participants of this study showed a relatively higher level of attitude towards aging and lower level of loneliness. Due to the traditional Chinese family philosophy [43], Chinese older people may coincidentally have both benefit and burden of taking care of their grandchildren. This may lead to a mixed influence on the older adults’ well-being. In this study, being the main caregiver of their grandchildren was negatively and significantly correlated with both positive attitudes toward aging and loneliness. Furthermore, a higher level of monthly income and living with spouse were positively and significantly correlated with positive attitudes toward aging. Such findings suggested that support from family members and sufficient financial resource could mitigate the negative impacts of caregiver burdens on well beings among Chinese older people [45]. In line with literatures [46], smoking was negatively correlated with positive attitudes toward aging and positively correlated with loneliness. Therefore, more attention should be given to current smokers in programs promoting healthy aging.

PA plays an important role in healthy aging. Given its positive effects on physiological health, independent mobility, cognitive function, and psychosocial wellness, older people are strongly encouraged to participate in PA [47, 48]. A review indicated that intergenerational program were promising approaches to reduce social isolation and help older people remain active, even for those with cognitive impairment (e.g., dementia) [49]. Therefore, co-participation in PA with grandchildren may be a new strategy to promote healthy aging with the concept of intergenerational activities. In this study, all five types of Co-PA with grandchildren are significantly and positively correlated with positive attitudes towards and better family harmony. Previous studies suggested that caring for grandchildren would improve aging attitudes and intergenerational support, which would hence increase life satisfaction among Chinese older adults [45]. A better life satisfaction and fulfilment of grandchildren caring through Co-PA could contribute to a better family harmony. Since this was a cross-sectional study and could not establish causal relationship, an alternative explanation was that older adults who had more positive attitudes toward their physical changes might be more confident about their physical capacity, and hence more likely to engage in Co-PA with their grandchildren.

This study has several limitations. First, we did not collect information from older adults who refused to join the study. It is possible that participants and refusals would have different characteristics. Self-selection bias existed. Second, due to the difference in socioeconomic status, healthcare system, and the availability of PA facilities, the findings might not be applicable to other cities in China. Shenzhen is one of the most developed cities in mainland China. It is expected the residents’ health literacy and availability of PA facilities in Shenzhen would be better than other less developed cities in China. Therefore, more older adults might engage in Co-PA with their grandchildren compared to their counterparts in less developed regions. Moreover, participants might over-report engagement with Co-PA, attitudes toward aging, and family harmony, and underreport loneliness due to social desirability. In addition, levels of Co-PA might be subject to recall bias. Furthermore, causality could not be established as this was a cross-sectional survey.

## Conclusions

In the Chinese context, Chinese people usually live in an extended family system including multiple generations. The current study provides the evidence for the positive correlations between Co-PA with grandchildren and their psychosocial outcomes among Chinese older people. Results support that grandparent-grandchildren Co-PA may serve as a new strategy for promoting healthy aging in Chinese older people. Health authorities might consider developing appropriate intergenerational PA programs.

## Supplementary Information

Supplementary Material 1. Shenzhen older adults health survey.

## Data Availability

The data that support the findings of this study are available from the corresponding author (Dr. Zixin WANG), upon reasonable request.

## Abbreviations

PA: Physical activity
Co-PA: Co-participation in physical activity
FHS: Family harmony scale
AAQ: 24-item attitudes to aging questionnaire
CI: Confidence intervals
SARS-CoV-2: Severe acute respiratory syndrome coronavirus 2
SD: Standard deviation
CDC: Center for disease control and prevention
