# Supplementary material for: Play together with grandchildren: a potential useful strategy for promoting healthy aging suggested by the evidence of 1,293 Chinese older adults
Source: BMC Geriatr. 2026 Apr 14;26:734. doi: 10.1186/s12877-026-07465-z (PMC13200448; doi:10.1186/s12877-026-07465-z)
Supplement: Supplementary file 1 — Supplementary Material 1. Shenzhen older adults health survey. [file 12877_2026_7465_MOESM1_ESM.docx]

**Shenzhen Older Adults Health Survey**

**Part 1: Background characteristics**

1-1 How old are you: _____ years

1-2 What is your gender: □_1_ Male □_2_ Female

1-3 What is your education level?

□_1_ Primary school or below □_2_ Junior high □_3_ Senior high or equivalent

□_4_ College □_5_ University □_6_ Postgraduate

1-4 What is your relationship status:

□_1_ Single □_2_ Married or cohabited with a partner □_3_ Divorced or separated □_4_ Widowed

1-5 What is your employment status?

□_1_ Retired □_2_ Full-time □_3_ Part-time □_4_ Freelance □_5_ Others, please be specific

1-6 What is your monthly personal income?

□_1_ Below 3000C NY □_2_ 3000-4999 CNY □_3_ 5000-6999 CNY □_4_ 7000-9999 CNY

□_5_ 10000-14999 CNY □_5_ 15000 CNY or above

1-7 Do you have the following chronic conditions?

|  | 有 | 没有 | 不清楚 |
| --- | --- | --- | --- |
| A) Hypertension | 1 | 2 | 3 |
| B) Other cardiovascular diseases | 1 | 2 | 3 |
| C) Chronic lung diseases | 1 | 2 | 3 |
| D) Chronic liver diseases | 1 | 2 | 3 |
| E) Chronic kidney diseases | 1 | 2 | 3 |
| F) Diabetes mellitus | 1 | 2 | 3 |
| G) Other diseases, please be specific: _____________ | | | |

1-8 Did you receive confirmed diagnosis of SARS-CoV-2 (received positive results through rapid antigen testing or nucleic acid amplification testing)?

□_1_ No □_2_ Yes

1-9 How many doses of COVID-19 vaccination did you receive?

□_1_ 0 dose □_2_ 1 dose □_3_ 2 doses □_4_ 3 doses □_5_ More than 3 doses

1-10 Have you received a seasonal influenza vaccination since September 2023?

□_1_ No □_2_ Yes □_3_ Uncertain

1-11 Did you smoke in the past year?

□_1_ No □_2_ Yes □_3_ Uncertain

1-12 Did you drink over five cans of beer, or five cups of Chinese wine or wine in on occasion in the past year?

□_1_ No □_2_ Yes □_3_ Uncertain

1-13 Are you living with anyone?

□_1_ Yes --- Please answer 1-13A □_2_ No --- Please answer 2-1

1-13A Who are you living with?

□_1_ Spouse □_2_ Children □_3_ Grandchildren □_4_ Other relatives □_5_ Domestic helpers

□_7_ Others, please be specific:__________________

**Part 2: Co-PA with grandchildren**

2-1 Do you have a grandchild? □_1_ No --- please answer 3-1 □_2_ Yes

2-2 How old is your grandchild/grandchildren?

□_1_ All of them were 18 years or above □_2_ Some of them were 18 years or above

□_3_ All of them were under 18 years old

2-3 Are you the main caregiver of your grandchild/grandchildren?

□_1_ No □_2_ Yes

2-3 How often did you perform the following activities with your grandchild/grandchildren?

|  | Never | <1 day per month | 2-3 days per month | 1 day per week | >1 days per week |
| --- | --- | --- | --- | --- | --- |
| 1) Walking or cycling together in the free time | 1 | 2 | 3 | 4 | 5 |
| 2) Playing sport together | 1 | 2 | 3 | 4 | 5 |
| 3) Participating sport competition together | 1 | 2 | 3 | 4 | 5 |
| 4) Going to the park, playground, beach or similar places together | 1 | 2 | 3 | 4 | 5 |
| 5) Going to an indoor recreation center together | 1 | 2 | 3 | 4 | 5 |

**Part 3 Menth health and psychosocial variables**

3-1Do you agree with the following statements?

|  | Strongly disagree | Disagree | Neutral | Agree | Strongly agree |
| --- | --- | --- | --- | --- | --- |
| A) It is important to take exercise at any age | 1 | 2 | 3 | 4 | 5 |
| B) Growing older has been easier than I thought | 1 | 2 | 3 | 4 | 5 |
| C) I don’t feel old | 1 | 2 | 3 | 4 | 5 |
| D) My identity is not defined by my age | 1 | 2 | 3 | 4 | 5 |
| E) I have more energy than I expected for my age | 1 | 2 | 3 | 4 | 5 |
| F) Problems with my physical health do not hold me back from doing what I want | 1 | 2 | 3 | 4 | 5 |
| G) My health is better that I expected for my age | 1 | 2 | 3 | 4 | 5 |
| H) I keep as fit and active as possible by exercising | 1 | 2 | 3 | 4 | 5 |

3-2 In the past 2 weeks, how often do you have the following feeling?

|  | Hardly ever or never | Sometimes | Often |
| --- | --- | --- | --- |
| A) Lack of companionship | 1 | 2 | 3 |
| B) Left out | 1 | 2 | 3 |
| C) Isolated | 1 | 2 | 3 |

3-3 Do you agree with the following statement related to your family?

|  | Strongly disagree | Disagree | Neutral | Agree | Strongly agree |
| --- | --- | --- | --- | --- | --- |
| A) My family functions well for all members | 1 | 2 | 3 | 4 | 5 |
| B) My family’s day-today interactions are peaceful | 1 | 2 | 3 | 4 | 5 |
| C) Family members accommodate each other | 1 | 2 | 3 | 4 | 5 |
| D) I am proud of my family | 1 | 2 | 3 | 4 | 5 |
| E) My family is harmonious | 1 | 2 | 3 | 4 | 5 |
